# Supplementary figures and images for: A flexible generative algorithm for growing in silico placentas
Source: PLoS Comput Biol. 2024 Oct 7;20(10):e1012470. doi: 10.1371/journal.pcbi.1012470 (PMC11486434; doi:10.1371/journal.pcbi.1012470)

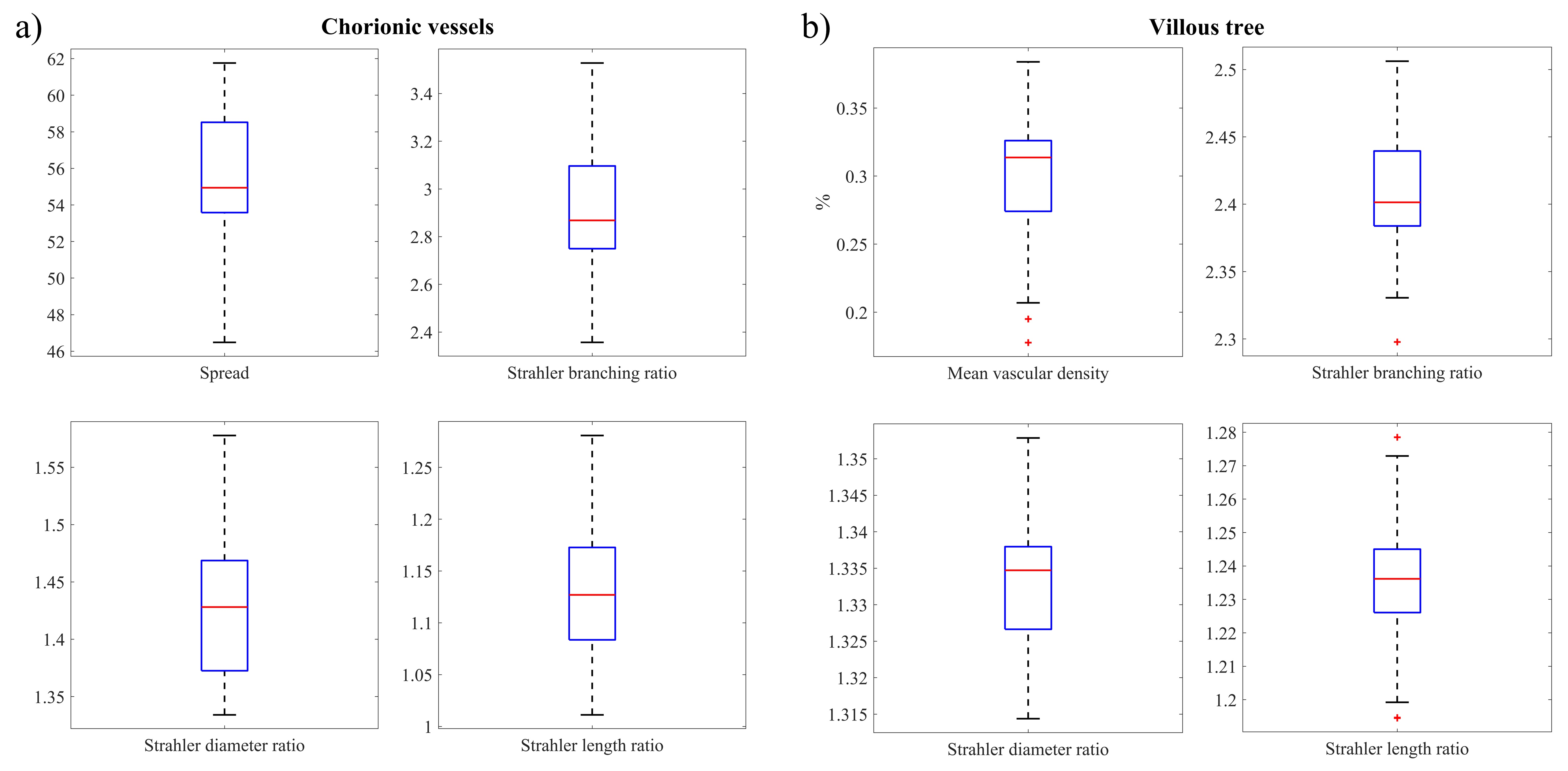

Supplement: S1 Fig — (TIF) [file pcbi.1012470.s001.tif]

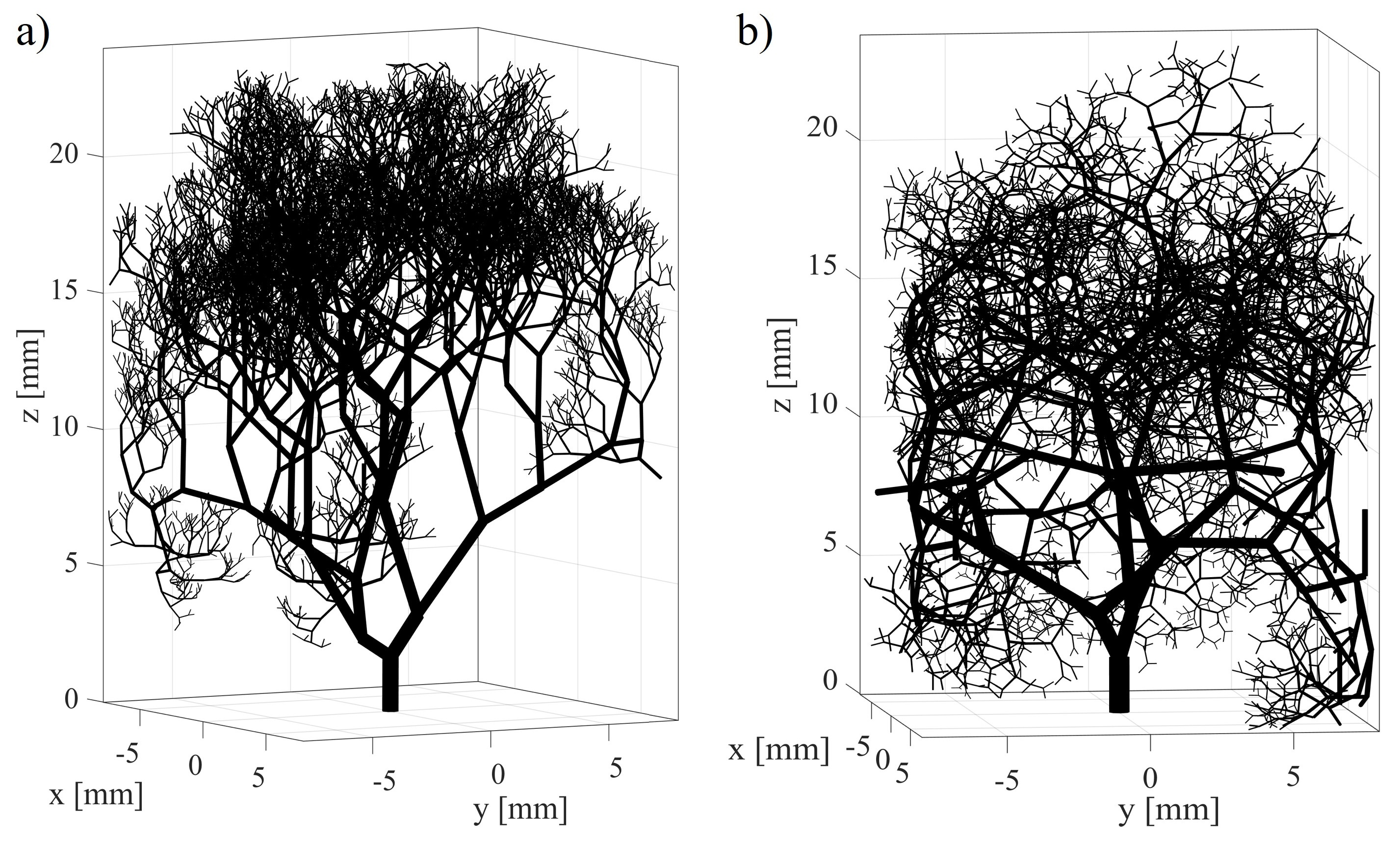

Supplement: S2 Fig — a) Fetal tree generated with cf1 = 0.4 and cf2 = 0.6. b) Fetal tree generated with cf1 = 0.8 and cf2 = 0.2. (TIF) [file pcbi.1012470.s002.tif]
