# Supplementary material for: A flexible generative algorithm for growing in silico placentas
Source: PLoS Comput Biol. 2024 Oct 7;20(10):e1012470. doi: 10.1371/journal.pcbi.1012470 (PMC11486434; doi:10.1371/journal.pcbi.1012470)
Supplement: S1 Table — The original chorionic plate surface mesh (1251 seeds) yields chorionic vascular structures with hindered topological metrics (e.g. lower number of chorionic vessels and less branching generations), while the mesh with sub-triangulated elements (4951 seeds) gives rise to better vascular structures (e.g. higher number of chorionic vessels with increased spread). (PDF) [file pcbi.1012470.s003.pdf]

	Key topological metrics				
**Number of seeds**	N. vessels	Mean branching gen.	Spread	Mean path length (mm)	Strahler b. ratio
1251	48	2.24±1.03	55.81	111.40±27.57	2.55
1251	38	2.36±1.33	50.81	87.01±18.48	2.48
4951	100	3.94±1.77	56.12	104.98±16.88	3.07
4951	83	3.74±1.94	60.59	99.37±25.15	3.00
