# Supplementary material for: A flexible generative algorithm for growing in silico placentas
Source: PLoS Comput Biol. 2024 Oct 7;20(10):e1012470. doi: 10.1371/journal.pcbi.1012470 (PMC11486434; doi:10.1371/journal.pcbi.1012470)
Supplement: S2 Table — Higher cf2 values result in fewer chorionic vessels, along with reduced spread and smaller mean path lengths. This is the result of vessels being generated towards the centroid of the chorionic plate. Higher cf1 values, on the other hand, promote greater vessel distribution across the chorionic plate, with an increased number of vessels and longer mean path lengths, though this can cause vessels to bypass the inner region of the chorionic plate. To achieve a balanced distribution of vessels while maintaining appropriate topological characteristics, a middle range of values is recommended (e.g. cf1 = 0.3–0.7; cf2 = 0.3–0.7). (PDF) [file pcbi.1012470.s004.pdf]

	Key topological metrics				
cf_1; cf_2	N. vessels	Mean branching gen.	Spread	Mean path length (mm)	Strahler b. ratio
0.1; 0.9	53	3.26±1.68	45.93	97.37±26.20	2.76
0.2; 0.8	95	3.92±1.73	53.52	93.49±22.85	2.61
0.3; 0.7	95	3.50±1.52	57.88	112.95±18.67	2.30
0.4; 0.6	100	3.56±1.48	56.66	111.26±20.59	2.60
0.5; 0.5	100	4.32±2.03	56.48	101.95±19.55	4.48
0.6; 0.4	89	3.56±1.56	59.31	104.56±16.13	2.66
0.7; 0.3	100	3.98±1.54	60.06	106.26±18.93	3.56
0.8; 0.2	91	3.85±1.88	61.04	101.86±20.46	2.54
0.9; 0.1	100	3.76±1.47	61.53	103.88±22.06	2.62
