# Supplementary material for: A flexible generative algorithm for growing in silico placentas
Source: PLoS Comput Biol. 2024 Oct 7;20(10):e1012470. doi: 10.1371/journal.pcbi.1012470 (PMC11486434; doi:10.1371/journal.pcbi.1012470)
Supplement: S3 Table — While smaller toll values are associated with lower computational times, they hinder vessel generation as highlighted by a smaller number of vessels and mean branching generations. In contrast, increasing toll values lead to appropriate topological metrics, but at a much higher computational cost. Therefore, a middle range toll (e.g. 1.5 mm, equivalent to 8–13% of chorionic vessel lengths) offers a balanced compromise between optimal topological metrics and computational efficiency. (PDF) [file pcbi.1012470.s005.pdf]

						Computational time (s)
	**Key topological metrics**					**Computational time (s)**
tol_l **(mm)**	N. vessels	Mean branching gen.	Spread	Mean path length (mm)	Strahler b. ratio	
0.1	18	1.09±0.88	45.03	86.45±15.98	3.18	2.26
0.4	60	2.84±1.14	54.74	100.74±26.74	2.43	125.63
0.8	74	3.60±1.87	53.07	102.13±22.82	2.91	702.00
1.2	98	4.38±1.90	55.12	101.36±26.83	3.54	809.86
1.5	100	4.15±1.87	57.34	108.57±17.37	2.60	851.00
1.8	100	3.79±1.55	54.88	99.49±24.24	2.73	915.70
2.2	100	3.77±1.53	55.64	99.78±16.06	2.67	1535.00
2.6	100	3.91±1.54	57.12	93.43±17.20	2.73	2462.41
3	100	4.16±1.70	56.15	100.60±20.99	2.64	3736.63
