# Supplementary material for: A flexible generative algorithm for growing in silico placentas
Source: PLoS Comput Biol. 2024 Oct 7;20(10):e1012470. doi: 10.1371/journal.pcbi.1012470 (PMC11486434; doi:10.1371/journal.pcbi.1012470)
Supplement: S4 Table — While smaller tolθ values are associated with lower computational times, they hinder vessel generation as highlighted by a smaller number of vessels. In contrast, increasing toll values lead to a greater number of generated vessels, but at a much higher computational cost. Therefore, a middle range tolθ (e.g. 0.2182 radians, equivalent to 25–35% of chorionic vessel parent-daughter branching angles) offers a balanced compromise between optimal topological metrics and computational efficiency. (PDF) [file pcbi.1012470.s006.pdf]

		Key topological metrics				Computational time (s)
tol_θ	N. vessels	Mean branching gen.	Spread	Mean path length (mm)	Strahler b. ratio	
0.0698	66	3.13±1.27	55.67	101.14±32.36	2.85	320.64
0.1221	79	3.39±1.67	55.13	102.90±20.90	3.29	277.46
0.1744	83	3.96±1.65	51.45	93.96±23.31	2.73	470.73
0.2182	100	3.68±1.43	55.95	98.37±16.48	2.29	672.77
0.2617	83	3.78±1.72	54.25	94.70±21.92	2.59	1013.99
0.3140	100	4.11±1.83	55.40	99.05±23.03	3.10	1778.67
0.3838	100	3.85±1.69	58.94	102.93±22.95	2.66	1385.23
0.4536	100	3.85±1.44	53.43	94.15±16.05	3.17	2705.44
0.5233	100	4.12±1.70	53.96	94.94±21.05	3.31	7237.84
