# Supplementary material for: A flexible generative algorithm for growing in silico placentas
Source: PLoS Comput Biol. 2024 Oct 7;20(10):e1012470. doi: 10.1371/journal.pcbi.1012470 (PMC11486434; doi:10.1371/journal.pcbi.1012470)
Supplement: S5 Table — cf1 and cf2 greatly affect the spatial distribution of vascular branches, as quantified by distances between vascular nodes and the basal plate (Distance 1) or between different vascular nodes (Distance 2). To obtain fetal trees with appropriate spatial dispersion, a middle range of values is recommended (e.g. cf1 = 0.5–0.7; cf2 = 0.3–0.5). (PDF) [file pcbi.1012470.s007.pdf]

	Key topological metrics			
**cf_1; cf_2**	Mean branching gen.	Strahler b. ratio	Distance 1 (mm)	Distance 2 (mm)
0.1; 0.9	13.24±1.83	2.69	3.66±1.56	5.52±2.49
0.2; 0.8	13.33±1.78	2.58	4.48±1.94	7.06±3.29
0.3; 0.7	13.42±1.70	2.56	4.95±1.50	7.33±3.41
0.4; 0.6	13.39±1.70	2.57	4.86±1.97	8.51±3.85
0.5; 0.5	13.44±1.65	2.42	9.72±4.61	10.19±4.60
0.6; 0.4	13.44±1.68	2.54	9.62±3.19	9.40±4.15
0.7; 0.3	13.44±1.67	2.49	10.59±3.85	9.91±4.12
0.8; 0.2	13.45±1.71	2.53	11.20±4.80	10.14±4.34
0.9; 0.1	13.12±1.83	2.57	13.61±4.63	10.64±4.30
