# Supplementary material for: A flexible generative algorithm for growing in silico placentas
Source: PLoS Comput Biol. 2024 Oct 7;20(10):e1012470. doi: 10.1371/journal.pcbi.1012470 (PMC11486434; doi:10.1371/journal.pcbi.1012470)
Supplement: S6 Table — While smaller tolθ values are associated with lower computational times, they hinder vessel generation as highlighted by fewer branching generations and lower vascular density. This is also quantified by distances between vascular nodes and the basal plate (Distance 1) or between different vascular nodes (Distance 2). Increasing toll values results in more branching generations, higher vascular density, and greater vessel spatial distribution, though at a higher computational cost. Therefore, a middle range tolθ (e.g. 0.1178 radians, equivalent to 15% of villous vessel parent-daughter branching angles) offers a balanced compromise between optimal topological metrics and computational efficiency. (PDF) [file pcbi.1012470.s008.pdf]

	Key topological metrics				Computational time (s)
tol_θ	Mean branching gen.	Mean vasc. density	Distance 1 (mm)	Distance 2 (mm)	
0.0157	8.11±3.08	0.16	9.86±3.89	7.96±4.63	1.80
0.0393	12.87±1.86	0.22	9.48±3.84	7.95±3.57	21.65
0.0628	13.32±1.72	0.17	11.52±3.91	9.26±4.22	65.08
0.0942	13.41±1.68	0.24	10.01±3.76	8.97±4.16	144.65
0.1178	13.36±1.72	0.22	9.62±3.19	9.40±4.15	135.53
0.1414	13.35±1.72	0.31	8.95±4.30	9.49±4.54	151.65
0.1728	13.48±1.65	0.28	8.99±3.21	9.47±3.89	163.55
0.2042	13.45±1.70	0.25	10.69±3.12	9.73±4.19	162.56
0.2356	13.44±1.66	0.27	10.14±4.15	9.40±4.35	172.67
0.3142	13.51±1.61	0.25	11.36±4.01	9.73±4.03	314.14
