# Supplementary material for: A flexible generative algorithm for growing in silico placentas
Source: PLoS Comput Biol. 2024 Oct 7;20(10):e1012470. doi: 10.1371/journal.pcbi.1012470 (PMC11486434; doi:10.1371/journal.pcbi.1012470)
Supplement: S7 Table — (PDF) [file pcbi.1012470.s009.pdf]

**Abbreviation**	**Meaning**
SA	Spiral artery
IVS	Intervillous space
FGR	Fetal growth restriction
MRI	Magnetic resonance imaging
3D	3-dimensional
ROI	Region of interest
EE	Elementary effects
KL	Kullback-Leibler Divergence
