# Supplementary material for: A flexible generative algorithm for growing in silico placentas
Source: PLoS Comput Biol. 2024 Oct 7;20(10):e1012470. doi: 10.1371/journal.pcbi.1012470 (PMC11486434; doi:10.1371/journal.pcbi.1012470)
Supplement: S8 Table — The general form is defined here, with some parameters being applicable to different scenarios (e.g. k can be associated with the generation of chorionic or villous vessels, becoming kc or kv, respectively). (PDF) [file pcbi.1012470.s010.pdf]

Parameter	Meaning
ltd	Length-to-diameter ratio
d	Diameter
l	Length
θ	Branching angle
k	Murray's Law bifurcation exponent
V	Placental volume
r_{maj}	Long placental radius
r_{min}	Short placental radius
E	Placental eccentricity
t_{half}	Half of placental thickness
CCI	Umbilical cord centrality index
mt	Minimum distance between cord insertion and chronionic plate periphery
A_{chor}	Chorionic surface area
d_c	Chorionic plate centroid
ud	Umbilical artery diameter
a	Asymmetry of branching generations
bg	Maximum number of branching generations
bn	Maximum number of segments
cf_1, cf_2	Global distribution penalty weights 1 and 2
n_p	Number of placentones
CC_r, CC_h	Central cavity radius and height
d_s	Stem diameter
D	Distance between candidate daughter node and other nodes/mesh domains
$\boldsymbol{\delta}$	List of candidate daughter nodes
$\mathbf{L}$	List of weighted scores for all tree nodes
DL	Line-to-line minimum distance
v	Vessel volume
μ^*	Absolute mean of elementary effects
σ	Standard deviation of elementary effects
