# Supplementary material for: A flexible generative algorithm for growing in silico placentas
Source: PLoS Comput Biol. 2024 Oct 7;20(10):e1012470. doi: 10.1371/journal.pcbi.1012470 (PMC11486434; doi:10.1371/journal.pcbi.1012470)
Supplement: Attachment — Submitted filename: Response to Reviewers.pdf [file pcbi.1012470.s011.pdf]

Decision on your article submitted to PLOS Computational Biology (PCOMPBIOL-D-24-00315)

Dear Dr C. de Oliveira,

Thank you very much for submitting your manuscript "A flexible generative algorithm for growing placentas" for consideration at PLOS Computational Biology. As with all papers reviewed by the journal, your manuscript was reviewed by members of the editorial board and by several independent reviewers. The reviewers appreciated the attention to an important topic. Based on the reviews, we are likely to accept this manuscript for publication, providing that you modify the manuscript according to the review recommendations.

Please prepare and submit your revised manuscript within 30 days. If you anticipate any delay, please let us know the expected resubmission date by replying to this email.

When you are ready to resubmit, please upload the following:

[1] A letter containing a detailed list of your responses to all review comments, and a description of the changes you have made in the manuscript. Please note while forming your response, if your article is accepted, you may have the opportunity to make the peer review history publicly available. The record will include editor decision letters (with reviews) and your responses to reviewer comments. If eligible, we will contact you to opt in or out
[2] Two versions of the revised manuscript: one with either highlights or tracked changes denoting where the text has been changed; the other a clean version (uploaded as the manuscript file).
Important additional instructions are given below your reviewer comments.

Thank you again for your submission to our journal. We hope that our editorial process has been constructive so far, and we welcome your feedback at any time. Please don't hesitate to contact us if you have any questions or comments.

Sincerely,

Alison Marsden
Academic Editor
PLOS Computational Biology

Pedro Mendes
Section Editor
PLOS Computational Biology

Response to the Editors:
Dear Editor in Chief,
We would like to thank you for your time taken in processing our manuscript and the reviewers for their feedback and comments. We have revised our manuscript according to the provided feedback and are resubmitting it again. Below we have addressed each point raised by the reviewers and have identified where this has led to a change in the manuscript (red highlights).

Best regards,
Diana Oliveira

Reviewer comments:

Reviewer #1:

This paper introduces a novel generative algorithm for creating in silico placentas. One of the advantages of the proposed algorithm is that it allows direct control over morphological parameters, a limitation of previous volume-filling algorithms.
There are some details relating to the validation of the algorithm that could be improved.

Response: We thank the reviewer for their positive assessment of the paper and for the feedback provided. We have edited the manuscript to provide additional information on the algorithm and the results presented. Any changes made to the text are highlighted in red, with the respective point in brackets at the end of the text. We address each point raised by the reviewer in more detail below.

R1P1. What is the rationale for the tolerance choice in the generation of the chorionic and villous vessels?

Response: We assume the reviewer refers to the tolerance choices for branch lengths and branching angles included in Equation 12. We chose the tolerance ranges reported in the manuscript through heuristic exploration and visual inspection of the generated vascular structures. We note that the tolerances can be user-defined in the algorithm, therefore, algorithm users can create different feto-placental vascular structures by adjusting these tolerances. Our primary objective when choosing tolerances was for the vessel generation to respect user-defined constraints (e.g. reaching the maximum number of generations), while obtaining a vascular structure with an appropriate number of vessels consistent with previous in silico models and in vivo/ex vivo observations. We observed that exceedingly low tolerances resulted in fewer selected candidate daughter nodes and generated vessels, leading to abrupt termination of vascular structures in both chorionic and villous vessels. In the case of chorionic vessels, lower branching angle tolerances hindered appropriate vessel spread throughout the chorionic plate. After exploring different tolerance values, we ultimately defined a branching angle tolerance range of 25-35%. This led to the selection of an adequate number of candidate daughter nodes, yielding appropriate chorionic vessel generation and spreading and resulting in a comparable chorionic vessel count to other studies. A narrower tolerance range (8-13%) for chorionic branch lengths proved effective for appropriate branch generation, particularly when paired with a higher branching angle tolerance. In the case of villous vessels, we determined that a fixed 15% tolerance in branching angles was sufficient to select enough candidate daughter nodes and lead to appropriate vessel generation.

We have edited the text explaining Equation 12 in Section “General notions of feto-placental vasculature generation” to improve clarity on our rationale behind the definition of the tolerances for the generation of chorionic and villous vessels (lines 270-281).

R1P2. The description of some of the rules for tree generation lacks a reference or rationale. For example,

Line 738: To account for empty space to be occupied by venous vessels, we assume that if DLij > 7/2 (di + dj), the generated daughter branch is accepted; otherwise, the algorithm proceeds to step 4.

Or

Line 729: The ROI is defined as a sphere of radius (3/5)ld,

Can the authors explain what this is doing exactly:
Line 744: The surface meshes are then converted to convex meshes, which represent collision geometries, and tested for collision using built-in functions from the Robotics System Toolbox.

Response: We agree that some descriptions of the algorithm could be improved. Regarding the specific examples mentioned above:

"Line 738: To account for empty space to be occupied by venous vessels, we assume that if DLij > 7/2 (di + dj), the generated daughter branch is accepted; otherwise, the algorithm proceeds to step 4."

There was a typo on the manuscript regarding the expression for the DLij threshold, as this should be in function of branch radius and not diameter. It should be DLij > 7/2 * (di/2 + dj/2) = 7 * (di + dj). This has been amended on the manuscript.

We have also included a rationale for how this expression is derived in the manuscript (lines 892-901). Briefly, for two arterial branches, the minimum DLij required to prevent branch-to-branch intersection (while still allowing branches to be in contact) should be equal to or above (di/2 + dj/2). If we account for empty space to be occupied by venous vessels, we adapt this threshold to include two venous branches (in addition to the arterial ones) with radii of 3/2*di/2 and 3/2*dj/2. These radii are derived as stated in Section "Model – Morphological metrics computed". The new threshold accounting for both arterial and venous vessels then becomes 5*(di + dj). However, and since we want to avoid branch-to-branch contact while allowing sufficient space for additional vessels, we assume DLij > 7*(di+dj).

"Line 729: The ROI is defined as a sphere of radius (3/5)ld"

We have edited the text corresponding to step 2 of "Computational implementation: Branch intersection checks" to improve clarity on our rationale behind the definition of the region of interest (ROI) for branch-to-branch intersections (lines 877-886). Briefly, only the branches with start/end nodes within this ROI are used on branch-to-branch intersection tests. This reduces the computational cost associated with this task. For each candidate daughter branch of length l^i, a spherical ROI of radius $x*l^i$ is generated, where x is a scaling factor. We determined x via heuristic exploration of different scaling factors and visual inspection of the generated vascular structure. A value of 3/5 was deemed suitable, as it allowed for an appropriate selection of branches while keeping a feasible computational cost and leading to intersection-free vascular structures.

“Line 744: The surface meshes are then converted to convex meshes, which represent collision geometries, and tested for collision using built-in functions from the Robotics System Toolbox.”

The collision test function employed in our pipeline is based on the Gilbert–Johnson–Keerthi (GJK) distance algorithm [REF1]. This algorithm computes the Euclidean distance between convex shapes to detect if they intersect or collide. We have included additional information about the detection of intersections/collisions in the manuscript (lines 905-918), as well as the main reference for the algorithm behind these.

R1P3. The point candidates for branching seem to be bounded to the initial mesh defined for the placental surface. How is this mesh generated? Does the point density (i.e., number of point candidates) affect the model's output? If so, how does it compare to the variabilities shown in Figure 11?

Response: To answer this question clearly, we have split it into three components below.

“How is the placental surface mesh generated?”

There are two surface meshes which determine the subsequent branching of chorionic vessels: the complete placental surface mesh and the chorionic plate mesh.

The complete placental surface mesh is generated from the 3D surface spheroid obtained using key morphological parameters (i.e. placental volume, long placental radius and placental eccentricity), as detailed in “Model”, subsection “Step-by-step description of placental size and shape”. This 3D surface spheroid is converted into patch format using a default MATLAB patch conversion function [REF2], with this patch structure containing the directly associated face and vertex data (which yield quadrangular mesh elements). The quadrangular mesh elements are then further subdivided to form triangular elements, yielding a triangulated surface mesh. This type of mesh is easier to use for subsequent steps of the pipeline and can be easily exported from MATLAB in typical mesh formats (e.g. stl). The text referring to the generation of the placental surface mesh has been edited to provide additional clarity (lines 219-222).

The chorionic plate mesh is obtained from the complete placental surface mesh defined above (“Model”, subsection “Step-by-step description of placental mesh and cord insertion”). The desired chorionic surface area is calculated using the equation for half of the area of an ellipsoid. This calculated value serves as the basis for extracting the corresponding chorionic surface mesh from the complete placental mesh. This process employs an iterative approach. Initially, the actual mesh surface area, which is the sum of the areas of all surface mesh elements, is estimated. This value is then compared iteratively with the desired surface area. While the actual surface area exceeds the desired one, rows of mesh elements are systematically removed, one at a time. The revised actual mesh surface area is then measured and compared with the desired surface area until they match. The final mesh surface area is set as the chorionic mesh surface. An additional line explaining this procedure has been included in the manuscript (lines 238-240). Its triangular mesh elements are further subdivided using a sub-triangulation function in MATLAB which creates one additional point per triangle edge, thus creating 4 final triangles per original triangle. This allows us to have sufficient seed points for the generation of chorionic vessels.

“Does the point density (i.e., number of point candidates) affect the model's output?”

The point density does affect the generation of chorionic vessels, especially the number of vessels outputted. However, we do not consider this point density to be a key user-input parameter to the pipeline, as we base our generative algorithm based on meaningful morphological parameters, supplemented by two global distribution penalty weights for fine-tuning of the spatial distribution of vessels. We heuristically explored different mesh densities and noticed that keeping the original mesh density from the placental surface mesh for the chorionic plate mesh (i.e. without further sub-triangulation) hindered chorionic vessel generation up to an appropriate number of branching generations, with less vessels being created. This was due to a lower amount of potential branching points available for chorionic vessels. By performing sub-triangulation of the chorionic mesh and therefore increasing point density, enough potential branching points are provided. This allows for chorionic vessels to spread throughout the chorionic plate, reaching an appropriate number of branching generations and yielding an appropriate number of vessels. The final point density is variable, being higher or lower depending on the size of the chorionic mesh surface. We have included additional explanation on this process in the manuscript, lines 240-242.

“If so, how does it compare to the variabilities shown in Figure 11?”

The variabilities in key metrics shown in Figure 11 correspond to 50 algorithm runs for chorionic and villous vessels (independently), where all runs have fixed input settings (Section “Analysis of algorithm outputs – Stochastic effects”, Table 2). In the case of chorionic vessels, all algorithm runs were performed with a fixed chorionic mesh point density. The reason for this is because this point density is not defined as an input parameter in the pipeline. The analysis of stochastic effects is focused on variability of model output between runs when applying key input parameters which rule the generation of chorionic vessels. As such, we assume that a formal comparison between the variabilities shown in Figure 11 and those obtained with different chorionic plate mesh point densities is out of scope of the paper and was not performed.

R1P4. Why is the placentone defined as a cuboid? An ellipsoidal or circular cylinder seems a more appropriate choice to describe this structure.

Response: For our study, we decided to follow the placentone definition in the computational study of maternal flow in a placentone by Saghian et al. [REF3], where a cuboid shape was chosen and parameterised. Effectively, defining the placentone as a cuboid both leads to a simpler mesh surface and an easier mathematical definition of a central cavity in comparison with an ellipsoidal surface. This choice also allows us to reduce the computational cost associated with generating a single fetal tree, especially considering the branch-to-mesh intersection checks performed at each algorithm iteration. We have included an additional explanation on this choice in the Discussion (lines 736-742).

Regardless, we agree with the reviewer’s comment that incorporating an ellipsoidal or semi-hemisphere placentone shape in the algorithm would be pertinent. In future iterations of our pipeline, we will consider adding an alternative placentone definition, providing users with the flexibility to select between a cuboid or semi-hemisphere shape.

R1P5. Some parameter ranges from the literature are missing in Table 4.

Response: We have edited this Table (now Table 5) and included the missing parameter ranges.

R1P6. About half of the parameters presented in Table 4 to evaluate the morphology of the generated trees are from in-silico studies (mostly one study; the other in-silico study mentioned is a quantification of micro-CT images). In the study from Clark et al. 2015, the authors use for validation, the placental vasculatures are generated using a volume-filling algorithm. According to the authors, the limitations of the volume-filling algorithm are precisely what motivates the generative algorithm presented in this paper. Hence, it seems contradictory to use results from the implementation of Clark et al. 2015 as validation for their tree generation algorithm. It is this reviewer's opinion that the authors should limit validation to in vivo or ex vivo data.

Response: We have found additional in vivo/ex vivo studies for validation of algorithm metrics and have updated Table 5 to incorporate these whenever applicable. Specific topological such as the maximum Strahler order and Strahler ratios are mainly calculated in in silico studies employing synthetic vasculatures representative of the human placenta (e.g. Clark et al. 2015 and Byrne et al. 2021 [REF4, REF5]). Other investigations into fetoplacental vascular structures, employing realistic geometries derived from micro-CT images, focus on mouse placentas [REF6, REF7], which we find impractical for validation purposes. As such, we believe that these specific metrics should still be compared against those obtained in previous in silico studies, as there is no other known ground truth, and these in silico studies are the only available (and feasible) source of comparison. We have included a sentence in the Discussion clarifying this (lines 659-661).

R1P7. What is the inner polygon of Figure 12 (and 14) representing?

Response: The inner polygon of Figures 12c and 14 denote the standard deviation linked with each metric, where applicable. Certain metrics, such as Strahler order and Strahler ratios, lack a standard deviation. On the other hand, metrics like mean branching generations and mean vascular density have a standard deviation, resulting in non-zero values for the inner polygon. We have removed these inner polygons representing the standard deviations as some parameters have zero standard deviation, which makes the plots confusing.

R1P8. The results highlight the crucial role of cf1 for the branching angle outputs. However, this metric is not reported in the literature, and it is provided by the authors to the model. How are the ranges reported in Table 1 defined? This is also not included in the discussion.

Response: The global distribution penalty weight 1 (or cf_1) is a user-defined parameter for which we provide a heuristic estimate. It is defined by us as part of a score function which fine-tunes the spatial distribution of feto-placental vessels. In the generation of chorionic vessels, cf_1 is a weight governing the distance to other vascular tree nodes, which is maximised. For the generation of villous vessels, this weight is linked to maximising the 3D space filled by these vessels, facilitating appropriate space division. In the same way that Cuntz et al. [REF8] defined their own cost function for the generation of neuronal branching structures inspired by Cajal’s laws of neuronal branching, we defined our penalty weights inspired by the vessel space-filling forces reported in Yang et al. [REF9], which aim to provide

a realistic spatial distribution of vascular structures. We included an additional sentence explaining the role of our penalty weights in the "Methods – Computational implementation: Fine-tuning spatial distribution of feto-placental vasculature" (lines 835-837).

The cf_1 ranges reported in Table 1 (0.4 – 0.8) were heuristically defined by exploring the effect of changes in cf_1 in 1) chorionic vessel generation and spread over the chorionic plate and 2) in the generation of a single fetal vascular structure. This explanation has been included in lines 418-421. Below we showcase two examples of fetal trees obtained with cf_1 fixed as 0.4 and 0.8. As displayed in Figure A a), a lower cf1 weight of 0.4 yields a fetal tree with branches being mainly directed towards the upper boundary (equivalent to the basal plate) and appearing to not spread as much within the placentone volume. This is due to the role played by a higher cf2 weight, which promotes minimisation of the distance between vascular branches and the basal plate. A higher cf1 weight of 0.8 (Figure A b)) allows for a greater maximisation of the distance between branches, with these spreading further within the placentone volume. We have included an explanation of how vessel spatial distribution changes with different cf1 weights in the Discussion (lines 665-673), and we include Figure A as a supporting figure in Supporting information (S2 Figure).

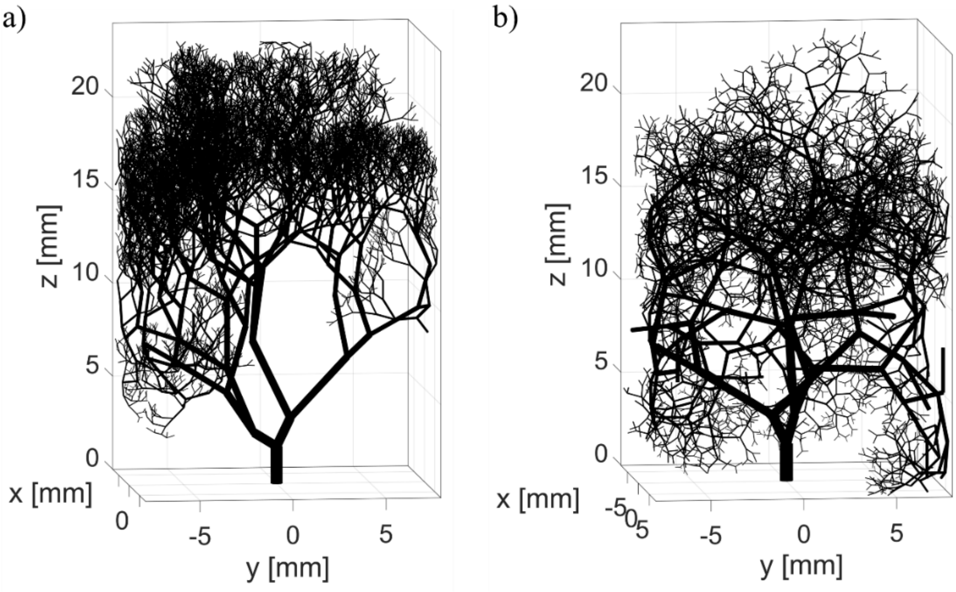

Figure A: Fetal trees generated with global branch distribution penalty weights a) cf1 = 0.4, cf2 = 0.6 and b) cf1 = 0.8, cf2 = 0.2.

R1P9. Can the authors provide additional details and interpretation of the results obtained regarding the "variability in output metrics from multiple algorithm runs"? What is exactly plotted in figures a) and b)? Is this divergence acceptable?

Response: We included additional details and interpretation of the results showed in Figure 11 a) and b) (lines 549-556, 558-561 and caption of Figure 11). The boxplots showed here represent the Kullback-Leibler (KL) divergence associated with key output metrics for chorionic (a) and villous (b) vessels in a healthy case. All output metrics included are represented by Gaussian distributions, and the KL divergence provides a measure of how similar two Gaussian distributions are: the lower the KL divergence value, the more similar two Gaussian distributions are. We ran the algorithm 50 times for

the generation of chorionic and villous vessels (independently), with sets of input parameters representing healthy vasculature. For each run, we obtained the Gaussian distribution associated each output metric. Using Equation 19, we can obtain the KL divergence, two Gaussians at a time, by performing combinations of Gaussians from the output space. For each output metric, if we consider a set of 50 Gaussians and retrieve 2 at a time, we obtain a total of 1225 combinations of Gaussians, yielding 1225 KL divergence values. The boxplots in Figure 11 a) and b) are a statistical representation of these 1225 KL divergence values for each output metric.

The KL divergence metric spans from 0 to +∞, providing a relative indication of variability across algorithm runs. No specific threshold defines the divergence as unacceptable. We performed additional experiments to answer R2P2 which show that the variability in output metrics obtained when generating a healthy tree is lower in comparison with that obtained when generating a dysfunctional tree. This is highlighted by much higher KL divergence ranges obtained for a tree representing fetal growth restriction (FGR) and for paired healthy-FGR fetal trees. We moved the information previously presented in Figure 11 c) and d) to the Supporting Information Section (S1 Figure), and the new KL divergence results are now represented in Figure 11 c) and d), as we believe these are more pertinent to the overall analysis. These new results suggest that it is not possible to generate a dysfunctional vascular structure when using input parameters associated with a healthy one. Moreover, they suggest that the variability in topological metrics is acceptable in the sense that the morphological parameters do not vary so much that they change the overall "type" of the fetal vasculature. Additional text interpreting these new results has also been added to Section "Analysis of stochastic effects – Variability in output metrics from multiple algorithm runs" (lines 566-577).

No other modelling studies of the placenta explore the KL divergence, and KL divergence results obtained by studies from other scientific fields are not valid for comparison against ours. Our analysis is focused on providing an overall sense of how stochastic our algorithm and associated metrics are, and we employ the KL divergence with this purpose. From our analysis, we observe that, for the generation of villous vessels, the mean path length stands out as the most stochastic output, while for the generation of chorionic vessels, multiple output metrics are stochastic (e.g. mean diameter of terminal arterial segments, mean terminal length, mean arterial branching generations).

R1P10. Not all the output variables included in Figures 7, 8, 9, and 10 seem to be included in Table 4. Also, the name used is different in some cases, which adds confusion.

Response: We have revised Figures 7-10 to align their parameter names with those listed in Table 5, wherever possible. It is worth noting that Table 5 presents metrics obtained for a complete feto-placental vascular structure, while Figures 7-10 relate to an assessment performed for chorionic vessels and villous vessels in a placentone, separately. While most of the output variables presented in Table 5 are also obtained for chorionic and villous vessels and presented in Figures 7-10, others were only computed for chorionic vessels (e.g. mean number of branches and spread) while one (vascular density) is computed for villous vessels in a placentone and assessed for the whole fetoplacental vasculature in a different Section of the Results ("Analysis of stochastic effects" – "Variability in vascular density: a two-case assessment").

R1P11. Figure 7and 9, add label in the color scale

Response: We have edited Figures 7 and 9 and included the color scale label.

R1P12. Image resolution of Figure 7, 8, 9,10, 11 and 13 is not optimal.

Response: We have revised Figures 7-11 and 13 and increased their resolution (dpi ≥ 400).

R1P13.

Line 322: missing word (Section")
Lines 349: missing words (more details in Sections "and")
Line 631: missing word (Section")

Response: We have read through the manuscript and added missing cross-references where necessary.

Reviewer #2:

The manuscript presents a flexible generative algorithm for creating synthetic feto-placental vasculature (arterial trees so far) with biophysical models and user-defined control. Compared with previous generating algorithms, a substantial advance of the algorithm presented herein is to allow direct control of the vasculature morphological parameters (e.g. vessel dimensions, branching angles) for designated feto-placental network characteristics reflecting healthy or pathological feto-placental structures, with a notable feature of stochastic variability for a given set of input parameters. The algorithm itself is introduced in detail with clear physiological underpinning and demonstrated through clinically-relevant examples, together with comprehensive quantification and sensitivity analysis. Overall, this is a well-motivated in silico framework and offers a new avenue for investigating the structure-function relationships in the placenta. With further developments to incorporate venous trees and complete capillary pathways, the framework can pave the way for more efficient organ-scale placenta modeling and inform diagnosis of placental disorders in the future. However, certain aspects of the manuscript need to be improved to enhance its clarity and impact, which are detailed below.

Response: We thank the reviewer for their positive assessment of the paper and for the feedback provided. We have edited the manuscript to enhance its clarity and impact. Any changes made to the text are highlighted in red, with the respective point in brackets at the end of the text. We address each point raised by the reviewer in more detail below.

R2P1. The stochastic nature of the algorithm is a desired feature to reflect biological variability but may also impose challenges on reproducibility. Does the algorithm have a mechanism (e.g. random seed generator) to reproduce a designated vasculature generated with a given set of user-defined parameters?

Response: Although the algorithm produces stochastic vascular structures, reproducibility is not compromised, as it is possible to exactly replicate a specific generated vasculature by fixing the seed

for MATLAB's random number generator (https://uk.mathworks.com/help/matlab/ref/rng.html). We checked this for the algorithm for the generation of placentone fetal trees by running it multiple times using the same random seed and the same set of user-defined parameters, and obtained the exact same vascular structures after multiple runs. A sentence on this has been included in the Discussion, lines 751-753.

R2P2. The authors should also discuss the implications of the modelled stochasticity in a biological context as the realistic vascularisation process of the feto-placental network is unlikely to be purely random but rather orchestrated through mechanical/chemical signals during pregnancy. Also, it would be nice if the authors could clarify how the level of stochasticity-induced structural variation compares to the absolute sample differences associated with pathologies versus physiology.

Response: The generation of the feto-placental network using our algorithm is not purely random – it follows statistical priors which are guided by literature on realistic feto-placental structures when possible. The stochasticity in the generation of vascular structures emerges from a combination of factors. For example, these factors include 1) the use of statistical distributions to define input parameters, 2) the fine-tuning of spatial distribution of vascular branches, and 3) the assessment of branch-to-mesh and branch-to-branch intersection and branch termination. This means that our modelled vascular generation is a result of both deterministic and random processes. We agree that the vascularisation process of the placenta is far more complex, including, as the reviewer mentions, mechanical/chemical signals during pregnancy. Whilst we have not directly modelled these, many of our algorithm choices mimic these processes indirectly. Future work could include improving the current pipeline to account for additional factors which drive vascular generation in the placenta. We have incorporated this in the Discussion section (lines 754-762).

To better understand differences in stochasticity-induced structural variations between healthy and dysfunctional fetal vasculatures, we decided to expand the analysis of Figure 11 to additionally compute the KL divergence of the output metrics in two cases: 1) for dysfunctional placentone fetal trees; 2) for pairs of healthy and dysfunctional placentome fetal trees. Case 1 assesses the variation of key output metrics in dysfunctional trees with respects to the KL divergence and case 2 assesses the variation of these metrics between healthy and dysfunctional vascular structures, to quantify how far apart these structures are. For this purpose, we used the same 50 fetal trees (corresponding to a healthy case) and corresponding output metrics as in Figure 11. For the dysfunctional tree, we used the input parameters for a placentone villous tree representing fetal growth restriction (FGR) from Table 4 and ran the algorithm 50 times to grow 50 dysfunctional fetal trees. For case 1, we re-ran the same analysis as for Figure 11, but with a set of 50 dysfunctional fetal trees; for case 2, we re-ran the same analysis as for Figure 11, but with 50 healthy and 50 dysfunctional fetal trees, only using pairs of healthy-dysfunctional output metrics.

We have edited Table 2 from Section "Analysis of algorithm outputs – Stochastic effects" to include the input parameters used to generate placentone fetal trees representative of fetal growth restriction, and Section "Analysis of stochastic effects – Variability in output metrics from multiple algorithm runs" to include additional analysis on these results (lines 566-577). We also moved the information presented in Figure 11 c) and d) to the Supporting Information Section (S1 Figure) and included in Figure 11 c) and d) the boxplots associated with the computed KL divergences for this specific analysis, as we believe these are more pertinent to the overall analysis. We can observe that the stochastic KL divergence for the output metrics associated with FGR fetal trees (Figure 11 c)) is higher in comparison

with that from Figure 11 b) for healthy fetal trees. This is especially true for the mean path length KL, where the upper quartile is above 2, whereas for the healthy fetal tree it is below 1 (as shown in Figure 11 b)). This suggests that there is more variability in topological metrics within dysfunctional fetal vasculatures when compared to healthy fetal vasculatures. Figure 11 d) displays even higher KL divergences for output metrics from paired healthy and FGR fetal trees. For instance, the lower quartile of KL divergences associated with mean path length, mean diameter of terminal segments and mean terminal length is above 6, while the upper quartile is beyond 20 for mean path length. This suggests that it is not possible to generate a dysfunctional vascular structure when using input parameters associated with a healthy one, further emphasizing the topological differences between healthy and dysfunctional fetal vasculatures.

R2P3. Does the algorithm support both dichotomous branching and monopodial branching (as shown in the Fig 12b schematic)? And if yes, does it have a preference for either branching mode in different scenarios, say non-central v.s. centralised umbilical artery insertion? I suspect that unlike the predicted outcome in Fig 12c (only marginal difference in the mean vascular density but not other topological metrics), the structural differences for non-central and centralised insertion in real placentas may be more severe? Or are the topological metrics adopted here robust enought to tell the structural difference?

Response: Our algorithm supports dichotomous and monopodial branching in the generation of chorionic vessels, although there is no preference for either branching mode in non-central vs centralised umbilical artery insertion. The chorionic vascular structures depicted in Figure 12 exhibited a proportion of monopodial branches amounting to 25% and 21% for central and non-central umbilical insertions, respectively. This additional result has been included in the Section "Analysis of stochastic effects – Variability in vascular density: a two-case assessment" (lines 583-587). It has been previously shown that a non-central umbilical insertion is associated with a higher number of monopodial chorionic branches, while a centralised umbilical insertion yields a chorionic architecture which is mostly dichotomous [REF10]. The implementation of preferential branching patterns associated with specific umbilical cord insertions remain an aspect to be improved in future code release versions and has now been included in the manuscript (lines 702-709).

As per the reviewer's observation, the topological metrics (especially branching statistics) we report for whole fetoplacental vasculatures with non-central vs centralised umbilical insertion remain mainly unaltered. We have edited Figure 12c to best showcase topological differences between the two vascular structures (even if marginal) by altering polygon maximum/minimum limits in the starplot. Regardless, our results agree with those presented in the computational study by Byrne et al. [REF5]. Two of the main structural differences between vasculatures with non-central and centralised umbilical insertion are 1) differences in proportions of arterial supply and 2) vascular density and variability in regional vascularization. For instance, Byrne et al. found a more unequal arterial supply in the case of non-central insertion (0.34:0.66 ratio) when compared to a centralised insertion (0.55:0.45 ratio). We observe a similar asymmetry in blood supply in our results (0.37:0.63 vs 0.48:0.52). In addition, Byrne et al. employed micro-CT to assess regional vascularization in placentas with centralised vs non-central insertion and noted that the latter has higher variability in regional vascularization, with relatively larger regions of lower density vasculature. This is reflected in a higher coefficient of variation in vascular density (86.1% vs 76.9%). Although the fetoplacental vasculatures observed in our study accurately represent variability in regional vascularization, this variability remains consistent between the structure with a non-central umbilical insertion and a centralized insertion (coefficient of variation in vascular density = 81.34% vs 82.12%, respectively). This information has been included in lines 613-614. To

further analyse such structural differences between vasculatures with different umbilical insertions and draw more specific inferences, we would need to generate an additional number of fetoplacental vascular structures and evaluate their regional vascular density. This explanation and suggestion for future analysis has now been included in the Discussion section of the manuscript (lines 687-701).

With regards to the chorionic vasculature by itself, we can describe additional topological metrics that showcase structural differences between centralised and non-central umbilical insertions. A previous ex vivo study observed differences in the number of branching generations required to reach placental margins from the umbilical insertion: around 4-6 generations for a centralised insertion and 5-8 generations for a non-central insertion [REF10]. Our results agree with these observations: a centralised insertion is associated with 3.85±1.71 mean branching generations, spanning 3-5 generations to reach the placental periphery. On the other hand, a non-central insertion is associated with 4.27±2.04 mean branching generations, requiring 4-7 generations to reach the placental periphery. The variances in branching generations manifest in the entire pathway from the umbilical cord to terminal vessels, showing a higher distance in the case of non-central insertion (125.09±28.84 mm) compared to centralised insertion (121.17±14.15 mm). This is coupled with increased variability in vessel pathways for a non-central insertion vasculature, as indicated by the increased standard deviation. Moreover, the vasculature with centralised insertion has a higher spread of chorionic vessels throughout the chorionic plate (66.92) when compared to the one with non-central insertion (58.44). Additional text detailing these topological metrics has been added to the Section “Analysis of stochastic effects – Variability in vascular density: a two-case assessment” to showcase differences in chorionic vasculature for non-central and centralised umbilical insertion (lines 596-608).

R2P4. The whole feto-placental vasculature or placentone villous tree is generated using morphological parameters known a priori in literature, but there seems no validation or evaluation of the generated in silico replica against the biological counterpart where the morphological parameters were drawn? Is there a way to achieve such validation at this stage without functional inspection, which I understand will need blood flow simulations in the generated network (therefore out of this paper’s scope) to compare with clinical imaging data (e.g. flow rate, oxygenation measurements)? It would also be nice to discuss how clinical phenotypes have been reproduced, e.g. hypo/hyper-branching patterns.

Response: The morphological parameters employed as inputs in the generative algorithm are obtained from multiple studies, spanning *ex vivo*, *in vivo* and, when required, *in silico* data. Therefore, there is not a complete biological counterpart available for us to directly assess our generated vascular structures against. As imaging modalities evolve (e.g. *ex vivo* synchrotron imaging), future assessments of the placenta will provide carefully drawn fetoplacental vascular structures and associated sets of key morphological parameters. This will provide a biological counterpart for additional structural validation. We have included this in the Discussion (lines 778-781). Functional inspection of the generated vasculatures (i.e. blood flow and transport simulations) is stated as part of the potential applications and future work section in the manuscript.

The vascular structures generated with our pipeline are parameterised by inputted morphological parameters and combinations of these. We do not directly model hyper/hypo-branching patterns to mimic specific clinical phenotypes; instead, topological changes on the vascular structure are induced by modified morphological parameters, such as in the case of the fetal tree associated with fetal growth restriction from Figures 14 and 15. Previous studies have shown that, in fetal growth restriction, villi are more poorly vascularised at the placental periphery, associated with impaired vascular branching

and density, leading to hypovascular regions [REF5, REF11]. Figure 15 showcases that for the case of modelled fetal growth restriction, where the fetal tree has higher density in the inward placentone volume, not spreading towards the periphery (poorly perfused region). We have incorporated this in the Discussion section (lines 710-719).

Other clinical phenotypes of interest may include: reduced oxygen delivery to the villi (e.g. due to poor maternal blood flow into the IVS), which leads to hyper-branching patterns as an adaptive response to increase fetal uptake [REF12, REF13]; diabetes, associated with a less mature feto-placental network with hyper-branching patterns at microvascular level [REF14]. Whilst we do not model these directly, we acknowledge that it is a desirable feature to be implemented in future code releases (e.g. additional user options to control branching patterns directly by decreasing/increasing the number of continuation nodes in a vascular network). We have incorporated this in the Discussion section (lines 790-797).

R2P5. More details and analyses about the healthy/pathological placentone fetal trees in Fig 15 should be given to showcase the potential workflow from medical imaging data to in silico placenta.

Response: We have now included additional details regarding the placentone fetal trees from Figure 15 (and especially the fetal tree associated with fetal growth restriction) in the manuscript (lines 635-636 and 710-719). Here, we link vascular architecture alterations that occur in FGR (observed in studies using micro-CT and casting corrosion techniques) to the altered vascular topology obtained in our own results.

Some figures and text also need to be polished:

R2P6. Fig 1, the zoomed-in are (black box) in (a) does not quite correspond to the lobule detailed in (b). Also in (b), may use normal arrows to replace the dot-line pointers for indicating the flow directions.

Response: We have edited Figure 1 and adjusted the black box in (a) to match the corresponding lobule in (b). We have also replaced the dot-line pointers in (b) by normal arrows.

R2P7. Fig 3, the use of numbered sections in the diagram is an issue as the sections in the main text are not numbered.

Response: We have edited Figure 3 and removed the numbered sections from the diagram.

R2P8. Fig 5, consider modifing the diagram flow to better reflect the decision tree from step 3 to step 6.

Response: We have modified the diagram from Figure 5 and included additional intermediate steps to provide more information on the decision-making process.

R2P9. Fig 6, the feto-placental vasculature is not fit to size and somehow mispositioned in the coordinate system.

Response: We agree that the chorionic plate and vessels were not fit to size with relation to the dimensions of the whole vascular structure in isometric view. This part of the figure has now been edited to match those dimensions. We have also added additional text to clarify which parts of the figure are represented in isometric view and in side view. We included an additional coordinate system for figure parts in side view to indicate their placement in the xz plane. An additional explanation was also added in the figure caption: "Figure components are depicted in isometric (*xyz* coordinate system) and side (*xz* plane) views.".

R2P10. Figs 8, 10-11, the line plots are blurred when printed out. Consider a darker line colour. Also in the captions of Figs 8 & 10, "Clusters... associated with higher mu* and lower sigma values, are circled in black," is "lower sigma values" the case?

Response: We have edited Figures 8, 10 and 11 to include thicker line plots and to ensure they have higher resolution (600 dpi). We agree with the reviewer's assessment of the clusters presented in Figures 8 and 10, as some of these clusters were not always associated with "lower sigma values". We have included an objective measure to determine these clusters in Section "Topological assessment and sensitivity analysis", within "Analysis of algorithm outputs" (lines 436-443). We normalise mu* and sigma for each input parameter and rank these based on a score which maximises mu* and minimises sigma, where mu* and sigma have the same weight (equation 18). The three input parameters with highest score are then selected as the most influential for each output parameter. Figures 8 and 10, as well as their respective captions, have been edited to change the clusters referring to these influential input parameters. The text which refers to these figures has also been edited to reflect the changes in highly influential parameters determined as per equation 18 (lines 509-514, 522-524, 530-540).

R2P11. Figs 12c, 14, explain the base coordinates of the radar plots, which are sometimes non-zero.

Response: The base coordinates of Figures 12c and 14 denote the standard deviation linked with each metric, where applicable. Certain metrics, such as Strahler order and Strahler ratios, lack a standard deviation, resulting in a base coordinate of zero for their respective radar plots. On the other hand, metrics like mean branching generations and mean vascular density have a standard deviation, resulting in non-zero base coordinates for their radar plots. As the presence of base coordinates may be confusing for the reader, we have removed them and updated Figures 12c and 14 accordingly.

R2P12. Double check all figure captions and make sure the concerned symbols are explained.

Response: We have revised all figure captions and provided a key for all symbols depicted in the corresponding figures.

R2P13. Currently, the main text is quite dense and sometime difficult to navigate due to a certain level of redundancy. The text may benefit from streamlining the contents in the Model, Results and Method sections.

Response: We appreciate that the Model and Methods sections are dense in places. However, we have only made a few small changes because we think that formatting the sections like this (with a certain level of redundancy) is the simplest way to communicate what we did to a reader who is interested in understanding exactly how our algorithm works, down to the finest details. We appreciate that some readers will not be interested in these algorithmic intricacies; We have therefore added a sentence at the start of the Model section that guides these readers to two key Figures and one section that will quickly enable them to understand our algorithm at a high level (lines 160-162).

R2P14. Table 3, double check "settings from 6-13." It is unclear to me how the parameters in table 3 lead to a round (rather than oval) chorionic plate in Fig 12a, b.

Response: We agree that the caption from Table 3 was confusing. To clarify the input settings applied in different scenarios, we have split this table in two, Tables 3 and 4: these refer to the input settings for the whole vasculature (Table 3) and to the input settings for the placentone and fetal tree (Table 4).

The shape of the chorionic plate depends on the definition of the placental size and shape. As stated in "Model – Step-by-step description of placental size and shape", the shape of the placenta is characterised by its volume (V), its long placental radius (r_{maj}) and its eccentricity (E), all of which are user-defined using statistical distributions. The placental surface can appear more rounded or oval depending on the interplay between these parameters: for example, for the whole fetoplacental vascular structures presented in Fig 12a, b, the placental eccentricity was defined as a normal distribution with mean 0.49 and standard deviation 0.17, as stated in Table 3. For the ex vivo placentas assessed in Pathak's study, placental eccentricity ranged from 0 (circular shape) to 0.91 (highly elliptical shape). Our final eccentricity values obtained for the placentas in Fig 12a, b were 0.54 and 0.30, respectively. This means that the placenta from Fig 12b is closer to a circular shape while the one from Fig 12a is slightly more elliptical. Higher eccentricity values would lead to more elliptical placental shapes.

R2P15. Page 18 line 454, provide references for "consistent with previous reports."

Response: We have provided references for this statement (line 484).

R2P16. Some cross-references are not working, e.g. missing section titles on page 13 lines 316 & 322, page 14, line 350, page 30 line 631.

Response: We have read through the manuscript and added missing cross-references where necessary.

List of references mentioned in Responses:

REF1. Gilbert, E.G., Johnson, D.W., and Keerthi, S.S. A fast procedure for computing the distance between complex objects in three-dimensional space. IEEE Journal on Robotics and Automation. 1988; 4(2): 193–203. doi:10.1109/56.2083.

REF2: MATLAB surf2patch function documentation, https://uk.mathworks.com/help/matlab/ref/surf2patch.html (Accessed: 16-05-2024).

REF3: Saghian, R., James, J.L., Tawhai M.H. et al. Association of placental jets and mega-jets with reduced villous density. Journal of Biomechanical Engineering. 2017; 139(5): 05001-05001. doi:10.1115/1.4036145.

REF4: Clark, A.R., Lin, M., Tawhai, M.H. et al. Multiscale modelling of the feto-placental vasculature. Interface Focus. 2015; 5(2): 20140078. doi:10.1098/rsfs.2014.0078.

REF5: Byrne, M., Aughwane, R., James, J.L. et al. Structure-function relationships in the feto-placental circulation from in silico interpretation of micro-CT vascular structures. J Theor Biol. 2021; 517(110630). doi:10.1016/j.jtbi.2021.110630.

REF6: Rennie, M.Y., Cahill, L.S., Adamson, S.L., Sled, J.G. Arterio-venous fetoplacental vascular geometry and hemodynamics in the mouse placenta. Placenta. 2017; 58: 46-51. doi:10.1016/j.placenta.2017.08.007.

REF7: Rennie, M.Y., Rahman, A., Whiteley, K.J. et al. Site-specific increases in utero- and fetoplacental arterial vascular resistance in eNOS-deficient mice due to impaired arterial enlargement. Biology of Reproduction. 2015; 92(2): 48, 1–11. doi:10.1095/biolreprod.114.123968.

REF8: Cuntz, H., Forstner, F. Borst, A. Hausser, M. One rule to grow them all: a general theory of neuronal branching and its practical application. PLOS Comput Biol. 2010; 6(8): e1000877. doi:10.1371/journal.pcbi.1000877.

REF9: Yang, J., Wang, Y. Design of vascular networks: a mathematical model approach. International Journal for Numerical Methods in Biomedical Engineering. 2013; 29(4): 515-529. doi:10.1002/cnm.2534.

REF10: Gordon, Z., Elad, D., Almog, R. et al. Anthropometry of fetal vasculature in the chorionic plate. J Anat. 2007; 211(6): 698-706. doi:10.1111/j.1469-7580.2007.00819.x.

REF11: Junaid, T.O., Brownbill, P., Chalmers, N. et al. Fetoplacental vascular alterations associated with fetal growth restriction. Placenta. 2014; 35(10): 808-815. doi:10.1016/j.placenta.2014.07.013.

REF12: Kingdom, J., Huppertz, B., Seaward, G., Kaufmann, P. Development of the placental villous tree and its consequences for fetal growth. European Journal of Obstetrics, Gynecology, & Reproductive Biology. 2000; 92(1):35–43. doi: 10.1016/S0301-2115(00)00423-1.

REF13: Roberts, H., Bourque, S.L., Renaud, S.J. Maternal iron homeostasis: effect on placental development and function Reproduction. 2020; 160(4): 65-78. doi:10.1530/REP-20-0271.

REF14: Carrasco-Wong, I., Moller, A., Giachini, F.R. et al. Placental structure in gestational diabetes mellitus. Biochim Biophys Acta Mol Basis Dis. 2020; 1866(2):165535. doi:10.1016/j.bbadis.2019.165535.
