# Supplementary material for: A flexible generative algorithm for growing in silico placentas
Source: PLoS Comput Biol. 2024 Oct 7;20(10):e1012470. doi: 10.1371/journal.pcbi.1012470 (PMC11486434; doi:10.1371/journal.pcbi.1012470)
Supplement: Attachment — Submitted filename: Response to Reviewers_rev2.pdf [file pcbi.1012470.s012.pdf]

Decision on your article submitted to PLOS Computational Biology (PCOMPBIOL-D-24-00315R1)

Dear Dr C. de Oliveira,

Thank you very much for submitting your manuscript "A flexible generative algorithm for growing in silico placentas" for consideration at PLOS Computational Biology. As with all papers reviewed by the journal, your manuscript was reviewed by members of the editorial board and by several independent reviewers. The reviewers appreciated the attention to an important topic. Based on the reviews, we are likely to accept this manuscript for publication, providing that you modify the manuscript according to the review recommendations.

The manuscript is almost ready, but we need you to address this issue: "The authors should include quantitative data supporting the heuristic determination of parameters and parameter ranges in the supplementary". Of course you should try to address other issues in the attached reviews as much as possible.

Please prepare and submit your revised manuscript within 30 days. If you anticipate any delay, please let us know the expected resubmission date by replying to this email.

When you are ready to resubmit, please upload the following:
[1] A letter containing a detailed list of your responses to all review comments, and a description of the changes you have made in the manuscript. Please note while forming your response, if your article is accepted, you may have the opportunity to make the peer review history publicly available. The record will include editor decision letters (with reviews) and your responses to reviewer comments. If eligible, we will contact you to opt in or out
[2] Two versions of the revised manuscript: one with either highlights or tracked changes denoting where the text has been changed; the other a clean version (uploaded as the manuscript file).
Important additional instructions are given below your reviewer comments.

Thank you again for your submission to our journal. We hope that our editorial process has been constructive so far, and we welcome your feedback at any time. Please don't hesitate to contact us if you have any questions or comments.

Sincerely,
Pedro Mendes
Section Editor
PLOS Computational Biology

Response to the Editors:
Dear Section Editor,
We would like to thank you for your time taken in processing our manuscript and the reviewers for their additional feedback and comments. We have revised our manuscript according to the provided feedback and are resubmitting it again. Below we have addressed each point raised by the reviewers and have identified where this has led to a change in the manuscript (red highlights).

Best regards,
Diana Oliveira

Reviewer's Responses to Questions

Reviewer #1:

The authors have provided additional details on the methods, which facilitate understanding their approach.

Response: We thank the reviewer for their positive assessment of the revised manuscript.

R1P1. The authors should include quantitative data supporting the heuristic determination of parameters and parameter ranges in the supplementary (e.g., tolerances, number of seeds, cf1, and cf2 …). These parameters have a critical role in successfully generating a physiological placental vasculature; supporting information on how they are determined will be essential for reproducibility.

Response: We agree that several parameters determined heuristically for our generative algorithm are critical in the generation of physiological placental vascular structures and that additional supporting information on how they are determined is relevant. To include quantitative data supporting our choices, we have performed an additional in silico experiment where we vary these parameters away from the chosen values and quantify how those changes affect the resultant vascular structures and their associated topological metrics. For some of these parameters, we have also analysed the computational time associated with generating the vascular structures, as that is an important component in our decision-making process.

<u>1. Chorionic plate: number of seed points for chorionic vessel generation</u>
We have included a table in the Supplementary Information (S1 Table) which states key topological metrics obtained for chorionic vessels generated with two different seed numbers: the one from the original chorionic plate surface mesh and the one obtained after sub-triangulation of mesh elements. We showcase that vessel generation is hindered when using the original number of seeds, associated with altered topological metrics, hence the need for a more refined surface mesh with greater number of seeds, obtained with sub-triangulation of mesh elements (lines 240-244). This explanation is also included in the caption for S1 Table (List of captions for supporting information files).

<u>2. Chorionic vessels: penalty weights *cf1, cf2*</u>
We have included a table in the Supplementary Information (S2 Table) which states key topological metrics obtained for chorionic vessels generated with different penalty weights <u>*cf1, cf2*</u>. Extreme values for penalty weights alter key topological metrics and, to achieve a balanced distribution of vessels while maintaining appropriate topological characteristics, a middle range of values is recommended. This explanation is also included in the caption for S2 Table (List of captions for supporting information files) and the table is referenced in line 677.

<u>3. Chorionic vessels: tol_l (Model - General notions of feto-placental vasculature generation)</u>
We have included a table in the Supplementary Information (S3 Table) which states key topological metrics obtained for chorionic vessels generated with different tolerances in branch lengths (tol_l). Lower tolerance values alter key topological metrics (lines 274-277), but increased values are associated with a higher computational cost. To achieve appropriate topological metrics at a suitable computational cost, a middle range tolerance value is recommended. This explanation is also included in the caption for S3 Table (List of captions for supporting information files).

4. Chorionic vessels: tol_θ (Model - General notions of feto-placental vasculature generation)

We have included a table in the Supplementary Information (S4 Table) which states key topological metrics obtained for chorionic vessels generated with different tolerances in parent-daughter branching angles (tol_θ). Lower tolerance values alter key topological metrics (smaller number of vessels generated, lines 274-279), but increased values are associated with a higher computational cost. To achieve appropriate topological metrics at a suitable computational cost, a middle range tolerance value is recommended. This explanation is also included in the caption for S4 Table (List of captions for supporting information files).

5. Villous vessels: penalty weights *cf1, cf2*

We have included a table in the Supplementary Information (S5 Table) which states key topological metrics obtained for villous vessels generated with different penalty weights *cf1, cf2*. Extreme values for penalty weights alter distances between vascular nodes and the basal plate, as well as distances amongst vascular nodes. To achieve a balanced distribution of vessels while maintaining appropriate topological characteristics, a middle range of values is recommended. This explanation is also included in the caption for S5 Table (List of captions for supporting information files) and the table is referenced in line 677.

6. Villous vessels: tol_θ (Model - General notions of feto-placental vasculature generation)

We have included a table in the Supplementary Information (S6 Table) which states key topological metrics obtained for villous vessels generated with different tolerances in parent-daughter branching angles (tol_θ). Lower tolerance values alter key topological metrics (fewer branching generations, lower vascular density, lines 274-277), but increased values are associated with a higher computational cost. To achieve appropriate topological metrics at a suitable computational cost, a middle range tolerance value is recommended. This explanation is also included in the caption for S6 Table (List of captions for supporting information files).

7. Villous vessels: Spherical ROI radius (Methods - Computational implementation: Branch intersection checks - Definition of region of interest)

We have included a justification of our chosen spherical ROI radius for branch-to-branch intersection in lines 888-893. Briefly, for each candidate daughter branch of length l, a radius of (3/5)*l ensures that the ROI fully encapsulates the candidate daughter branch. This is mathematically sufficient to determine branch-to-branch intersections with any branches in proximity while keeping a suitable computational cost. A larger spherical ROI would encompass additional branches unnecessarily, resulting in increased computational times.

R1P2. On the topic of validation, this reviewer finds that validation with an in silico-generated vascular model may be insufficient. This is especially true in this case since the motivation for this new algorithm, according to the authors, is to address the limitations of previous in silico placenta vasculature generation algorithms.

References to the model's ability to represent psychological vascular trees should be limited to references that provide histological or image quantification of ex vivo data. For example, the sentence: "Both chorionic vessels and villous tree structures are strongly asymmetric in branching, as given by Strahler branching ratios beyond 2.3, within the range of those presented in the literature [20]" is presented as a fact, while the citation refers to a modeling study. In such cases, the authors should only claim that their model produces results similar to those of the cited research, making it clear to the reader that both are computational results.

Response: We agree that the validation of our in silico vascular model should be performed against studies including histological or image quantification of *ex vivo* data, and that sole comparison against other *in silico* studies is not sufficient to validate our computational predictions. We have ensured that *ex vivo* and *in vivo* data are used as the main source of validation of our computational metrics, using *in silico* predictions as the final source of comparison when no other data is currently available (as now included in lines 469-474). This case occurs, for example, for Strahler numbers (maximum Strahler ratio, Strahler diameter ratio and Strahler length ratio), which have not been previously obtained for *ex vivo* and *in vivo* placental data, hence the need for comparison against *in silico* studies (as presented in Table 5). As mentioned in the Discussion - Potential applications and future perspectives (lines 780-787), future quantitative assessments of the placenta using comprehensive imaging techniques (e.g. synchrotron computed tomography) will be able to provide additional sets of key morphological parameters and topological metrics such as Strahler numbers for additional structural validation of our fetoplacental vasculatures.
We have decided to still include all sources of comparison in Table 5 when available (e.g. *ex vivo* and *in silico* data for a certain topological metric), as we believe it is relevant to provide the reader with multiple benchmarks. We have also revised the manuscript to ensure that comparisons between our model results and those of other computational studies are clearly stated, ensuring that our results are not presented as a fact. As per the reviewer's suggestion, we toned down the text referring to Strahler branching ratios and the resulting vascular structure asymmetry (lines 682-684).

R1P3. Although computational cost is mentioned several times in the manuscript, no quantitative data is provided in this respect. Please include a short comment referring to the hardware employed and the compute time required to generate the full vascular tree. If available, compare against previously published algorithms for vascular generation.

Response: We have added a new section in the Manuscript (Methods - Computer specifications and algorithm performance, lines 931-952) which includes: 1) the computational specifications of the hardware used to generate and analyse all vascular structures; 2) the computational time required to generate the vascular structures (placentone fetal tree, chorionic vessels and complete fetoplacental vasculature); 3) a comparison against previous algorithms for vascular generation. We note that the computational performance of our algorithm is improved in comparison with previous algorithms for cerebrovasculature generation and those targeting complex anatomies. We also recognise that there is

room for improvement in future algorithm releases to reduce our computational time even further to attain performances similar to those of accelerated constrained constructive optimisation techniques.

Reviewer #2:

R2P1. The authors have satisfactorily addressed my comments and the manuscript is significantly improved in this revision. I would recommend publication with an optional suggestion for the authors to consider: adopt consistent axial scales between the gridlines for each parameter in Figs. 12 and 14 to facilitate date interpretation.

Response: We thank the reviewer for their positive assessment of the paper and for the feedback provided. We would like to mention that the axial scales for Figures 12 and 14 were selected to best showcase differences between cases and for each individual metric, hence the differences in scale. Whilst we thank the additional optional suggestion to adopt consistent axial scales in these Figures, such a change would make it more difficult for the reader to easily observe differences between cases.
